# Discovery of a conserved translationally repressive upstream open reading frame within the iron-deficiency response regulator *IDEF2*

**DOI:** 10.1186/s12870-024-05473-y

**Published:** 2024-09-30

**Authors:** Oscar Carey-Fung, Jesse T. Beasley, Ronan C. Broad, Roger P. Hellens, Alexander A. T. Johnson

**Affiliations:** 1 School of BioSciences, The University of Melbourne, Parkville, VIC 3010 Australia; 2 Department of Animal, Plant and Soil Sciences, School of Agriculture, Biomedicine and Environment, La Trobe University, Bundoora, VIC 3086 Australia; 3 GenXtraits, Alameda, CA USA

**Keywords:** Biofortification, CPuORF, Dual luciferase, IDEF1, Rice, Transcription factor, uORF, Wheat

## Abstract

**Background:**

Iron (Fe) deficiency affects 30–50% of the world’s population. Genetic biofortification of staple crops is a promising strategy for improving human nutrition, but the number of effective precision breeding targets for Fe biofortification is small. Upstream open reading frames (uORFs) are cis-regulatory elements within the 5’ leader sequence (LS) of genes that generally repress translation of the main open reading frame (mORF).

**Results:**

We aligned publicly available rice (*Oryza sativa* L.) ribo-seq datasets and transcriptomes to identify putative uORFs within important Fe homeostasis genes. A dual luciferase assay (DLA) was used to determine whether these uORFs cause repression of mORF translation and pinpoint LS regions that can be mutated for mORF derepression. A translationally repressive uORF region was identified in two positive regulators of the Fe-deficiency response: IDEF1 and IDEF2. The IDEF2-uORF peptide was highly conserved among monocots and a mutation series in the 5’ LS of the wheat (*Triticum aestivum* L.) *TaIDEF2-A1* gene demonstrated variable mORF derepression.

**Conclusions:**

Together these results reveal a possible regulatory mechanism by which IDEF2 transcription factors modulate the Fe deficiency response in monocots, and highlight novel precision breeding targets to improve crop nutrition and abiotic stress tolerance.

**Supplementary Information:**

The online version contains supplementary material available at 10.1186/s12870-024-05473-y.

## Highlight

We searched for translationally repressive uORFs within Fe homeostasis genes to find precision breeding targets for improved crop nutrition and discovered uORFs in *IDEF1* and *IDEF2*.

**Supplementary Information:**

The online version contains supplementary material available at 10.1186/s12870-024-05473-y.

## Introduction

Iron (Fe) homeostasis in plants is maintained by a network of transcription factors that regulate the expression of Fe chelators, transporters, and storage proteins. Nicotianamine is an Fe chelator that facilitates long-distance Fe transport within all higher plants and functions as a precursor to the secreted phytosiderophore 2’-deoxymugineic acid (DMA) in graminaceous monocots [1]. In the rhizosphere, Fe-DMA complexes are absorbed into roots by the yellow stripe-like (YSL) transporters [2, 3]. Knockout or knockdown of *nicotianamine synthase* (*NAS*) or *DMA-synthase* (*DMAS*) genes results in reduced sensitivity to Fe deficiency and reduced plant growth [4, 5], while overexpression of the rice *OsNAS2* gene increases grain Fe concentrations in rice and bread wheat (*Triticum aestivum* L.) without affecting agronomic performance [6, 7].

The expression of Fe homeostasis genes is regulated by several basic helix-loop-helix (bHLH) transcription factors, such as iron related transcription factor 2 and 3 (IRO2, IRO3) and positive regulator of Fe homeostasis 1, 2 and 3 (PRI1/2/3) [8–12]. Knockout or knockdown of positive regulators *OsIRO2* and *OsPRI1*/*2*/*3* in rice causes hypersensitivity to Fe deficiency, whereas overexpression of *OsIRO2* or *OsPRI2* genes upregulates the Fe deficiency response, enhances stress tolerance, and increases grain Fe concentration between 1.5- to 2-fold relative to wild-type (WT) plants [9, 11–13]. By contrast, knockout or overexpression of the negative regulator *OsIRO3* both result in hypersensitivity to Fe deficiency [8, 14–16]. Other positive regulators of the Fe deficiency response include the IRONMAN (IMA) family, and overexpression of an artificial *IMA* gene in rice increased grain Fe concentration 2-fold, whereas silencing the *IMA* gene family in Arabidopsis led to inhibition of Fe uptake [17, 18]. The haemerythrin motif-containing really interesting new gene (RING) and zinc finger proteins (HRZ1 and HRZ2) sense Fe levels and target bHLH transcription factors for degradation and attenuation of the Fe deficiency response, and knockdown of *OsHRZ2* in rice increases grain Fe concentrations up to 3.8-fold [19]. Two positive regulators of the Fe deficiency response in monocots are iron-deficiency-responsive element (IDE) binding factor 1 (IDEF1), a member from the ABI3/VP1 transcription factor family, and IDE binding factor 2 (IDEF2), a member of the NAC transcription factor family [20, 21]. The IDEF1 and IDEF2 transcription factors bind to IDEs in the promoter regions of several Fe homeostasis genes and upregulate their expression [20–22]. The expression of both *OsIDEF1* and *OsIDEF2* is constitutive in rice and conserved under differing environmental Fe conditions, and OsIDEF1 is capable of binding Fe ions directly [23, 24]. By contrast, the mechanism by which IDEF2 is modulated for IDE binding is unknown [20, 25].

Upstream open reading frames (uORFs) starting within the 5’ leader sequences (LS) of genes are cis-regulatory elements that post-transcriptionally regulate the main ORF (mORF). The proportion of uORF-containing transcripts in plant species ranges from 6 to 48%, predicted by the presence of AUG sequences upstream (uAUG) of the main ORF translation start site (mAUG) or upstream translation detected by ribosomal profiling [26, 27]. Conserved peptide uORFs (CPuORFs) are uORFs likely to produce peptides with metabolic functions, and despite a large number of well characterised CPuORFs within the literature, non-conserved peptide uORFs form the majority of all uORFs [28]. Although some uORFs have been shown to increase translation of the mORF, most uORFs act to attenuate translation as a noise reduction and energy saving measure [29, 30]. Mutating uORFs generally increases translational efficiency of the mORF thereby making them a valuable gene editing target for manipulating gene expression and crop breeding, however uORF identification remains a major hurdle [31]. The predominant methods for identifying plant uORFs include homology-based approaches (uORFinder, uORFSCAN, BAIUCAS/ESUCA), ribosomal profiling (psORF), and peptidogenomics (psORF) [28, 32–35]. In a rare case, a GWAS proteomic approach identified several natural uORF variants within maize (*Zea mays* L.) that altered protein abundance [36]. Of these existing methods, ribosomal profiling is the favoured method for uORF identification, which uses deep sequencing on ribosome protected fragments to provide an unbiased snapshot of translation events and allows the detection of cryptic translation events in non-canonical ORF sites [37].

In this study, we describe the search for translationally repressive uORFs within monocot Fe homeostasis genes as precision breeding targets and the discovery of uORFs in *IDEF1* and *IDEF2*. The *IDEF1*-uORF is only present in rice and does not respond to changes in environmental Fe whereas the *IDEF2*-uORF peptide is conserved amongst most monocots and responds to changes in environmental Fe. These translationally repressive uORFs represent useful targets for novel breeding efforts aimed at improving crop Fe nutrition and/or abiotic stress tolerance.

## Materials and methods

### Construction of ribosomal profiles

A ribo-seq dataset from NCBI was extracted and aligned with a rice (*Oryza sativa* L. cv. Nipponbare) cDNA dataset (Ensembl Plants) to identify non-canonical translation events outside of coding regions [38]. In this dataset, the rice plants were grown under control or salt stress conditions, but only the control ribo-seq dataset was used for the ribo-seq and transcriptome alignments. The ribo-seq datasets were quality checked (FastQC), trimmed to remove adapters and low-quality reads (TrimGalore) and aligned with the concatenated cDNA dataset (BWA-MEM) in Galaxy Australia (https://usegalaxy.org.au/) [39]. Extractions of ribosomal profiles for genes were performed using Rstudio (https://rstudio.com/ v4.2.1) with the following packages: Biostrings v2.64.1, fansi v1.0.4, Rcpp v1.0.10, seqinr v4.2.30, dplyr v1.1.2, Biostrings v2.64.1, ggplot2 v3.4.2, tidyverse v2.0.0, datapasta v.3.1.0. Approximately 35 members of important Fe homeostasis gene families (e.g. master regulators, bHLH transcription factors, and downstream targets) were searched in the ribo-seq dataset, however only 20 genes and their splice variants were identified and had high enough resolution to generate ribosomal profiles [40, 41]. A gene encoding an ascorbate biosynthesis enzyme, GDP-L-galactose phosphorylase (*OsGGP*), with a well characterised CPuORF was included as a positive control [42, 43]. Start codons, open reading frames (stop-to-stop), and transcript models were annotated based on the Rice Genome Annotation Project (http://rice.uga.edu/).

### Dual luciferase vector construction

A CaMV 2 × 35 S promoter from the pJIT60 vector was cloned into the pGreen II 0800-LUC vector upstream of the firefly luciferase coding sequence using *KpnI* and *XhoI* [44]. Leader Sequences were either PCR amplified from synthesised sequences (Twist Bioscience, South San Francisco, CA, USA and Azenta, Burlington, MA, USA), or PCR amplified from genomic DNA using Phusion^®^ High-Fidelity DNA Polymerase (NEB, Ipswich, MA, USA) and cloned downstream of the CaMV 2 × 35 S promoter and upstream of the firefly luciferase coding sequence using *NcoI* and *SalI*. Ligated plasmids were validated using a diagnostic restriction digest (*KpnI* and *ApaLI*) and sanger sequencing (primer sequence: GCCTTATGCAGTTGCTCTCCA). Mutated LSs were either synthesised, or produced by mutating WT sequences using the Q5^®^ Site-Directed Mutagenesis Kit (NEB, Ipswich, MA, USA). All Fe homeostasis genes that were suggested to contain uORFs by the psORF database (http://psorf.whu.edu.cn/#/) were included in the Dual Luciferase Assay (DLA) regardless of ribosomal profiling (Fig. S2). Putative uORF start codons (as predicted by PsORF http://psorf.whu.edu.cn/) within the m1 LS were mutated to either TAG, TAA or TGA. The four ATG codons that were identified using ribosomal profiling and sequence analysis within the *OsIDEF1* m2 LS were mutated to either TAG, TAA or TGA.

**Fig. 1:**
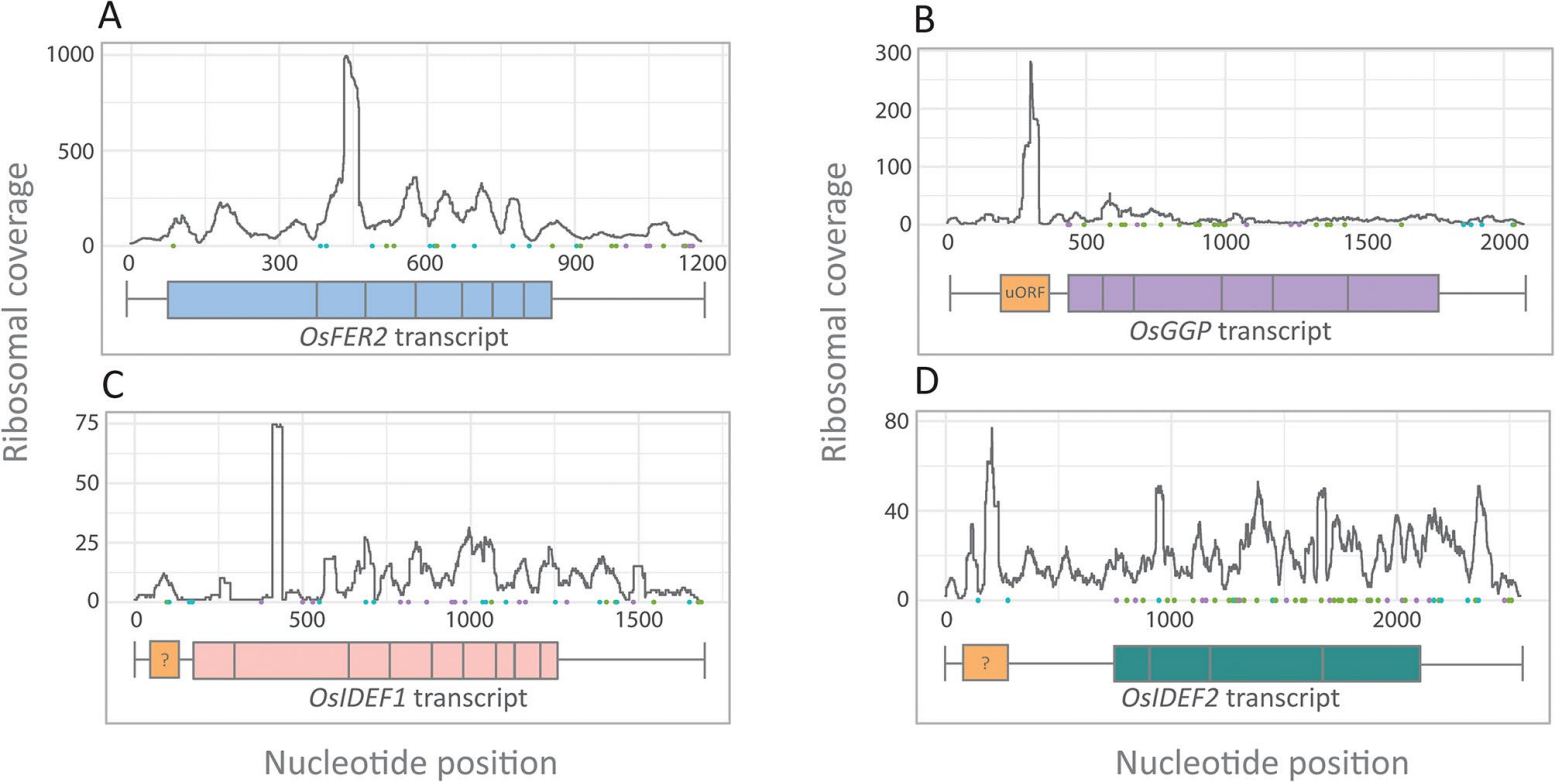
Ribosomal profiles of rice transcripts involved in nutrient homeostasis. The **(A)** *OsFER2*, **(B)** *OsGGP*, **(C)** *OsIDEF1*, and **(D)** *OsIDEF2* transcripts show varied ribosomal coverage within the 5’ LS, corresponding with the likelihood of non-canonical translation. Canonical ATG start codons within translation frame 1 (green), 2 (blue), and 3 (purple) are represented as dots along the x-axis. The transcript models are represented below the ribosomal profile with the 5’ LS (left-hand horizontal line), coding sequences (coloured boxes), 3’ UTR (right-hand horizontal line), and putative uORFs indicated within the 5’ LS (orange box)

**Fig. 2:**
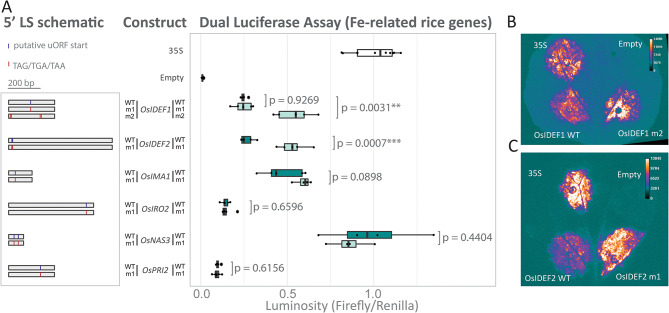
Dual luciferase assay of various Fe homeostasis 5’ LS from rice. **(A)** Schematic (left panel) of the WT or mutated (m1/m2) 5’ LS that were fused upstream of the firefly luciferase coding sequence and downstream of a 35 S promoter. Putative canonical and non-canonical start codons are indicated (blue line) and stop codons (red line) within the 5’ LS are indicated. A box plot (right panel) compares luminosity ratios (firefly/renilla) of the WT 5’ LS (dark green) and their mutated version (light green). The p-values compare the mutant and WT LS of the same gene as determined by a two-sample Students t-test assuming unequal variance (*n* = 5). Each biological replicate comprised of three leaf discs (averaged) from three infiltration sites on a single leaf. The luminosity ratios (firefly/renilla) were normalised to the 35 S vector (positive control containing no 5’ LS). The empty vector contains the firefly luciferase coding sequence with no promoter. Visual representation of an independently infiltrated *N. benthamiana* leaf containing the 35 S infiltration (top left), empty infiltration (top right) and **(B)** *OsIDEF1* WT (bottom left) and *OsIDEF1* m2 (bottom right) or **(C)** *OsIDEF2* m1 (bottom left) and *OsIDEF2* m1 (bottom right)

**Fig. 3:**
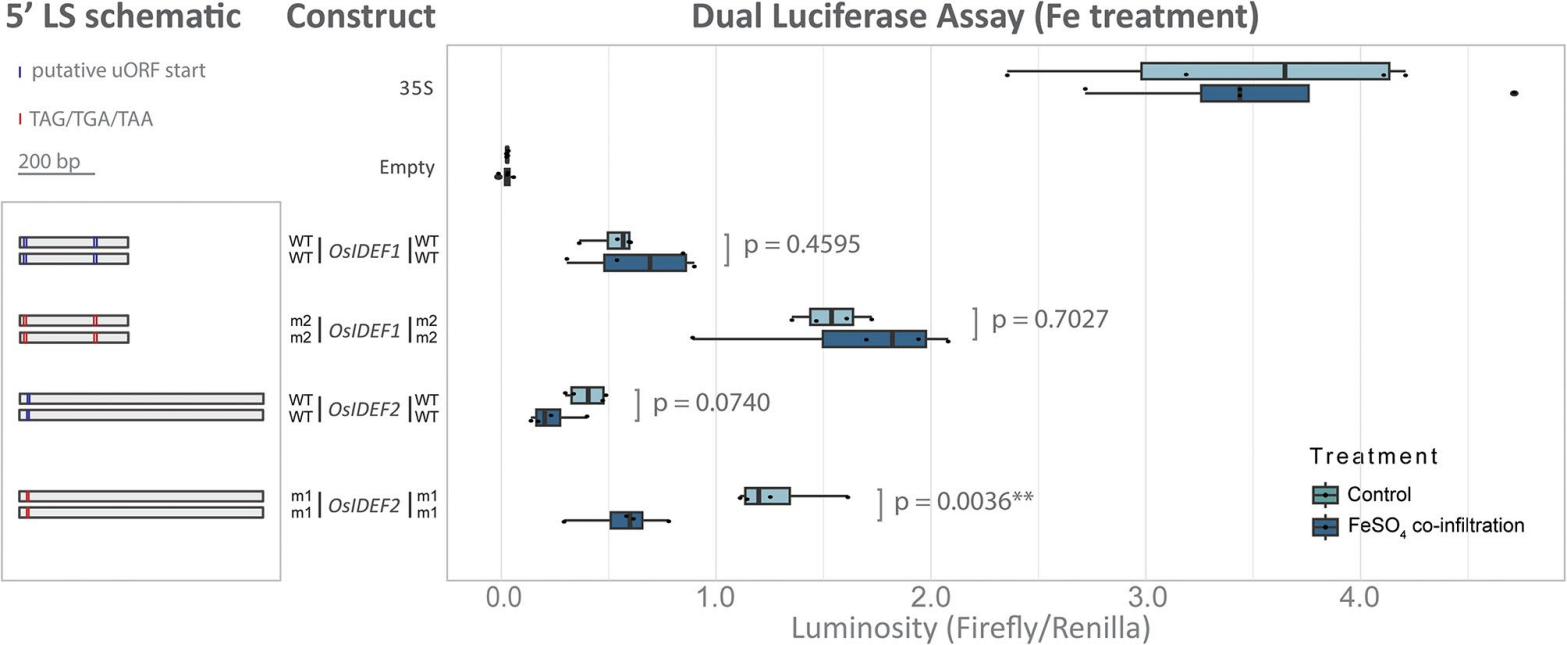
Dual luciferase assay of 5’ LS from rice. Schematic (left panel) of the WT or mutated (m1/m2) 5’ LS that were fused upstream of the firefly luciferase coding sequence and downstream of a 35 S promoter. Putative canonical and non-canonical start codons are indicated (blue line) and stop codons (red line) within the 5’ LS are indicated. A box plot (right panel) compares luminosity ratios (firefly/renilla) of the various 5’ LS as either the control single infiltrations (light blue) or co-infiltrations with FeSO_4_ (dark blue) to perturb Fe homeostasis. The p-values compare the control and co-infiltrated 5’ LS as determined by a two-sample Students t-test assuming unequal variance (*n* = 4). Each biological replicate comprised of three leaf discs (averaged) from three infiltration sites on a single leaf. The luminosity ratios (firefly/renilla) were not normalised to the 35 S vectors to demonstrate that FeSO_4_ co-infiltration does not interact with the luciferase genes without the addition of Fe-related 5’ LS

**Fig. 4:**
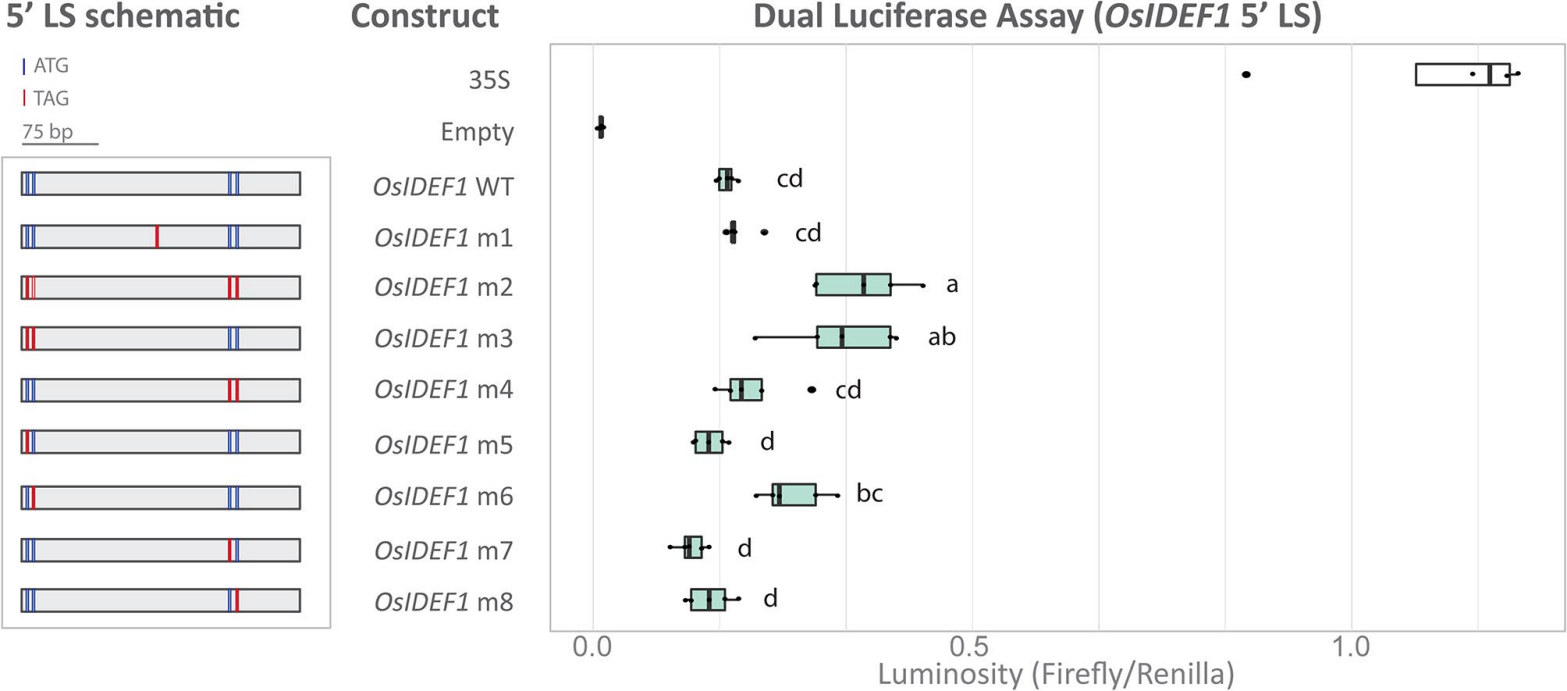
Dual luciferase assay of *OsIDEF1* 5’ LS from rice to identify the uORF start site or sites. Schematic (left panel) of *OsIDEF1* 5’ LS containing combinations of mutated ATGs that were fused upstream of the firefly luciferase coding sequence and downstream of a 35 S promoter. Putative canonical start codons are indicated (blue line) and stop codons (red line) within the 5’ LS are indicated. A box plot (right panel) compares luminosity ratios (firefly/renilla) of the various 5’ LS. Letters indicate significant differences (*p* < 0.05) between all LS (excluding the controls) as determined by a one-way ANOVA (post hoc Tukey’s HSD test, *n* = 5). Each biological replicate comprised of three leaf discs from three infiltration sites on a single leaf. The luminosity ratios (firefly/renilla) were normalised to the 35 S vector (positive control containing no 5’ LS)

### Agroinfiltration

**Fig. 5:**
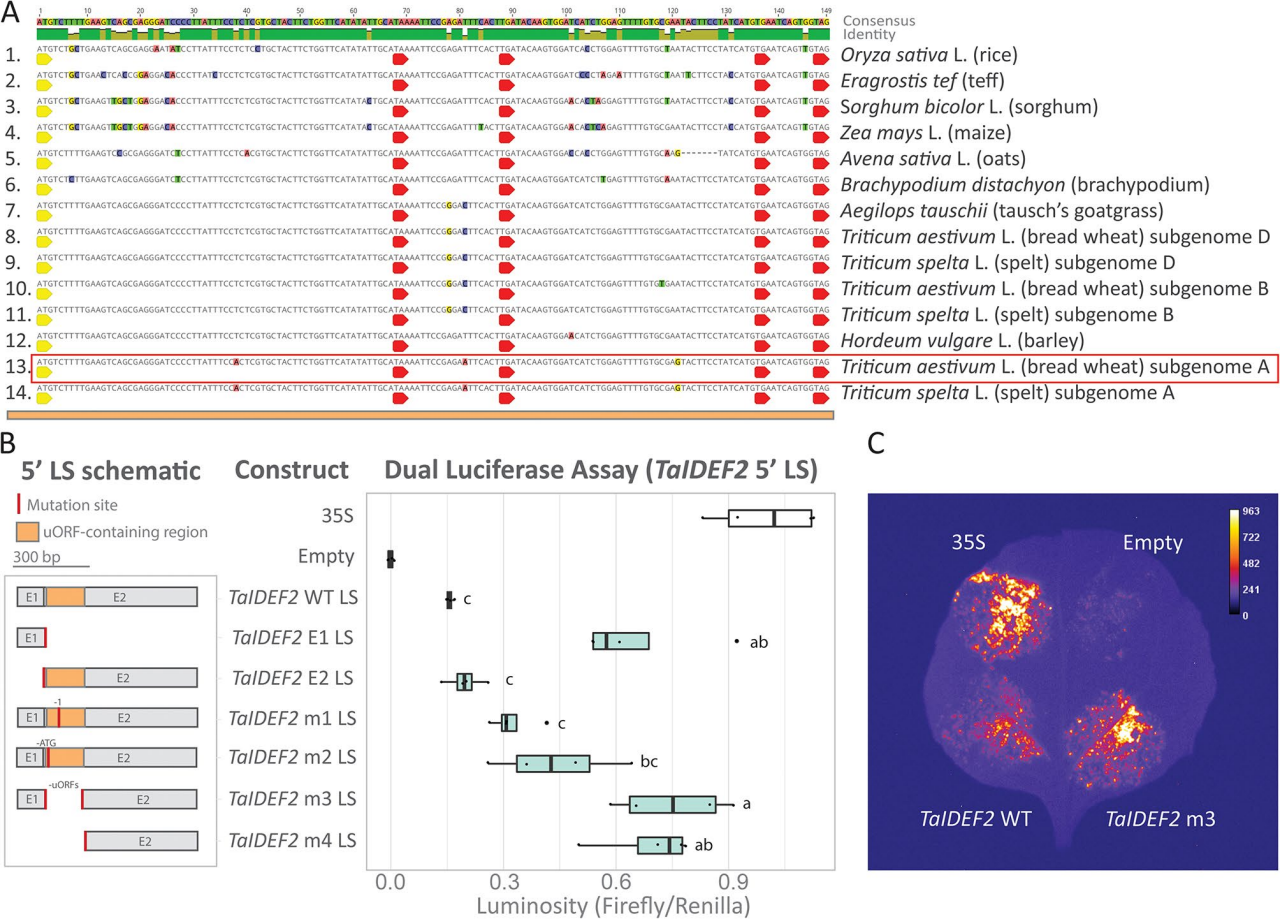
Dual luciferase assay of *TaIDEF2* 5’ LS from bread wheat. **(A)** Nucleotide alignment of the uORF-containing region amongst *IDEF2* orthologous sequences in monocots. The uORF-containing region located within the 5’ end of E2 was identified based on ribosomal peaks in Fig. 1D and analysis of conserved ORFs (stop-to-stop) within the 5’ LS. A conserved canonical start codon (yellow arrow) as well as conserved stop codons (red arrow) are indicated as possible start/stop sites of uORFs. The red rectangle indicates the 5’ LS used for the DLA. **(B)** Schematic (left panel) of the WT or mutated 5’ LS that were fused upstream of the firefly luciferase coding sequence and downstream of a 35 S promoter. The *TaIDEF2* 5’ LS comprises of two exons (E1 and E2). The uORF-containing region is indicated (orange box). A box plot (right panel) compares luminosity ratios (firefly/renilla) of the various 5’ LS. Letters indicate significant differences (*p* < 0.05) between all LS (excluding the controls) as determined by a one-way ANOVA (post hoc Tukey’s HSD test, *n* = 4). The luminosity ratios (firefly/renilla) were normalised to the 35 S vector (positive control containing no 5’ LS). **(C)** Visual representation of an independently infiltrated *N. benthamiana* leaf containing the 35 S infiltration (top left), empty vector infiltration (top right), *TaIDEF2* WT (bottom left), and *TaIDEF2* m3 (bottom right)

The reporter plasmids were co-transformed via electroporation into *Agrobacterium tumefaciens* (GV3101) with the helper plasmid pSOUP [44]. Single colonies were grown in liquid media overnight at 28 C°, washed in agroinfiltration solution (10 mM MgCl_2_ and 10 mM MES), and resuspended in agroinfiltration solution containing acetosyringone (200 µM) (Sigma-Aldrich, St. Louis, MO, USA). The cultures were left to incubate (gentle shaking) at room temperature in the dark for at least two hours and then normalised to 0.2 OD_600_ in a 10 mL volume. Each construct was infiltrated into the underside of six leaves of 4-week-old *Nicotiana benthamiana* plants (three leaves per plant) with four infiltration sites per leaf. Plants were grown in growth cabinets at 22 C° with a 16-hour light period. To determine if the Fe homeostasis uORFs responded to exogenous Fe supply (Fig. 3, S3), the agroinfiltration solution was normalised to 0.4 OD_600_ in a 5 mL volume and then mixed (1:1) with 5 mL of FeSO_4_ (0.2%). The photographed leaves (Figs. 2B and C and 5C) were independently infiltrated with a different construct in each quarter of the leaf (underside of leaf segment). DualGlo^®^ Luciferase Reagent was sprayed onto the underside of the leaf, left for ten minutes and photographed (Promega, Madison, WI, USA).

Bread wheat (*Triticum aestivum* L., cv. Fielder) was hydroponically grown for 2 weeks at 16 C° (8 h) and 21 C° (16 h). The solution contained NH_4_NO_3_ (0.2 mM), KNO_3_ (5 mM), Ca(NO_3_)_2_∙4H_2_O (2 mM), MgSO_4_∙7H_2_O (2 mM), and KH_2_PO_4_ (0.1 mM), and micronutrients H_3_BO_3_ (10 µM), MnCl_2_∙4H_2_O (5 µM), ZnSO_4_∙7H_2_O (5 µM), CuSO_4_∙5H_2_O (0.5 µM), NaMoO_4_∙2H_2_O (0.1 µM), and NaFe_3_ + EDTA (50 µM) (Sigma-Aldrich, St. Louis, MO, USA). Leaves (*n* ≥ 3) were infiltrated in a 10 cm area with the agroinfiltration solution. After seven days of growth, a firefly luciferin was infiltrated at the same agroinfiltration site and immediately photographed with a ten-minute exposure. The images were adjusted and analysed in ImageJ (version 1.54 g). Mean grey values were calculated within a 2496-pixel rectangle around the infiltration site. A Student’s Two-tailed T-test assuming equal variance was used to determine statistically significant differences between the *TaIDEF2* WT and *TaIDEF2* m3 infiltrations.

### Dual luciferase assay

Five days after infiltration, four or five of the six *N. benthamiana* leaves were chosen for the DLA (*n* = 4 or 5). Leaf discs (5 mm in diameter) were taken from areas adjacent to three of the four infiltration sites (representing three technical replicates). Leaf discs were placed in 100 µL of PBS buffer within a 96-well flat-bottom plate and ground with Tungsten Carbid Beads (Qiagen, Hilden, Germany) using the Geno/Grinder^®^ (SPEX SamplePrep, Metuchen, NJ, USA) or the TissueLyser II (Qiagen). Background luminosity was measured using a GloMax^®^-Multi Detection System or Cytation 5 Cell Imaging Multimode Reader (BioTek, Winooski, VT, USA). Luminosity was measured using the DualGlo^®^ Luciferase Assay System (Promega, Madison, WI, USA). For firefly luminosity, 75 µL of DualGlo^®^ Luciferase Reagent was added to the wells and incubated (gentle shaking) at room temperature for 10 min, then luminosity was measured using the plate reader. For renilla luminosity, 75 µL of DualGlo^®^ Stop & Glo^®^ Reagent was added to the wells and incubated (gentle shaking) at room temperature for 10 min, then luminosity was measured using the plate reader. Background luminosity readings were subtracted from firefly and renilla luminosity readings. The firefly luminosity values were normalised against the renilla luminosity. The three technical replicates were then averaged to form one biological replicate and the ratios were normalised against the 35 S construct (excluding Fig. 3, S3) to get the final normalised luminosity ratios.

### Statistical and phylogenetic analysis

Statistically significant differences were determined by a Student’s Two-tailed T-test assuming unequal variance (between two 5’ LS) or a one-way ANOVA with Tukey’s HSD test of multiple comparisons (amongst more than two 5’ LS) and calculated using Rstudio software (https://rstudio.com/ v4.2.1). A generalised linear model was used to determine the effect of Fe co-infiltration and mutation (Fig. 3). Dual luciferase assay (DLA) graphs and ribosomal profiles were generated using the ggplot2 v3.4.2 software package in RStudio (https://ggplot2.tidyverse.org/).

The *IDEF1* and *IDEF2* orthologous genes were identified using reciprocal blasts with *OsIDEF1* and *OsIDEF2* in Ensembl Plants (https://plants.ensembl.org/) and Rice Genome Annotation Project (RGAP, http://rice.uga.edu/). If available, the 5’ LS was annotated using Ensembl Plants, otherwise 200 bp upstream and 700 bp upstream of the translation start site for *IDEF1* and *IDEF2*, respectively, was used. Alignments were performed in Geneious (https://www.geneious.com/v11.0.18) using the MUSCLE alignment tool. Phylogenetic construction was performed using the PhyML v3.3.20180621 Geneious plugin with the LG substitution model and a bootstrap value of 1000 [45].

## Results

### High resolution peaks in the 5’ LS suggests non-canonical translation events

Of the 20 Fe homeostasis genes and their splice variants analysed we found evidence of translation events upstream of the mAUG in 9 genes (Table S1, Fig. S2). The *OsFER2* transcript is an example of a transcript with high ribosomal coverage but no evidence of translation in the 5’ LS (Fig. 1A). The *OsGGP* transcript contains a large peak where the uORF has previously been annotated (Fig. 1B). The *OsIDEF1* and *OsIDEF2* transcripts show mild and strong evidence of translation upstream of the mORF, respectively (Fig. 1C, D). Neither *OsIDEF1* nor *OsIDEF2* have previously characterised uORFs.

### The *OsIDEF1* and *OsIDEF2* 5’ LS contain translationally repressive uORFs

Of the nine genes that exhibited ribosomal peaks within the 5’ LS, six (*OsIMA*, *OsIRO2*, *OsNAS3*, *OsPRI2*, *OsIDEF1*, and *OsIDEF2*) contained uORF start sites or conserved ORFs in the 5’ LS that were testable using a DLA (Fig. 2). For the mutated (m) *OsIMA1* m1, *OsIRO2* m1, *OsNAS3* m1, and *OsPRI2* m1 sequences, the putative uORF start site was determined by the psORF database and then mutated to a stop codon (TAG, TGA, or TAA) (Fig. 2A). These mutated 5’ LS did not show statistically significant differences in mORF translation efficiency relative to WT sequences, indicating that no translationally repressive uORF is present at the selected non-canonical start site (Fig. 2A). The *OsIDEF1* m1 sequence was a mutated non-canonical start codon predicted by psORF, which did not show differences relative to the WT sequence. However, when all four canonical start codons (ATGs) within the 5’ LS were mutated into stop codons (*OsIDEF1* m2), translation of the mORF increases by 2.18-fold (*p* = 0.0031). We identified a region of high conservation within the 5’ LS of *OsIDEF2* orthologs. Within the conserved region is an ATG and a non-canonical start codon (CTG) predicted by psORF which were both mutated into stop codons (*OsIDEF2* m1), leading to a 1.99-fold increase in mORF translation (*p* = 0.0007). Firefly luminescence was elevated in *N. benthamiana* leaf segments infiltrated with *OsIDEF1* m2 and *OsIDEF2* m1 sequences relative to WT sequences (Fig. 2B and C).

### The *OsIDEF2*-uORF but not *OsIDEF1*-uORF responds to changes in environmental Fe

To determine if the *OsIDEF1* and *OsIDEF2* uORFs respond to changes in environmental Fe, we infiltrated *N. benthamiana* leaves with the Agrobacterium infiltration solution alone containing the DLA plasmids (control) or the Agrobacterium infiltration solution mixed with 0.2% FeSO_4_ (1:1) (co-infiltration) (Fig. 3). A generalised linear model found no effect of Fe treatment on the WT or mutated *OsIDEF1*-uORF sequences (p = 0.448) and a major effect of Fe treatment on the WT and mutated *OsIDEF2*-uORF sequences (p = 0.002). Under the control condition, luminosity of the *OsIDEF2* WT sequence was 1.69-fold higher (p = 0.074) and luminosity of the *OsIDEF2* m1 was 2.26-fold (p = 0.004) higher relative to the Fe condition. The *OsIMA1* LS showed no evidence for the presence of a translationally repressive uORF but showed an effect of Fe treatment (p = 0.002), suggesting that other factors could be regulating expression of *OsIMA1* via the 5’ LS (Fig. S3).

### The *OsIDEF1* 5’ LS contains a three amino acid non-conserved uORF

To identify which ATGs are the start site of a translationally repressive uORF within the *OsIDEF1* 5’ LS we created a series of *OsIDEF1* 5’ LS with different combinations of mutations (Fig. 4). Mutating the first two ATGs into stop codons (*OsIDEF1* m3) resulted in a 1.85-fold derepression in mORF expression relative to the WT sequence (p_adjusted_ = < 0.001). Mutating the second ATG alone (*OsIDEF1* m6) resulted in a 1.49-fold derepression in mORF expression relative to the WT sequence (p_adjusted_=0.059) indicating that it may be the main ribosomal binding site. These ATGs were not conserved in other *IDEF1* orthologs (Fig. S4A). However, another upstream ATG (uATG) was conserved in teff, sorghum, maize, brachy, oats, barley, rye, bread wheat, and spelt (subgenome A). We determined whether this uATG start site initiated translation of a repressive uORF by using the *TaIDEF1-B1* homoeolog in bread wheat for the DLA (Fig. S4B). The mutated sequence (*TaIDEF1* m1) did not alter translation of the mORF compared to the WT sequence (*p* = 0.818) indicating that it is not the start site of a translationally repressive uORF.

### The *IDEF2*-uORF is well conserved amongst monocots and can be mutated to fine-tune translation of the mORF

The uORF-containing region in the *IDEF2* is highly conserved amongst monocots (Fig. 5A, S6) but not eudicots. We identified a closely related, but not necessarily orthologous, gene *AtNAC082* (At5G09330) in *Arabidopsis thaliana*, with strong ribosomal peaks in the 5’ LS (Fig. S2AB). To assess if the *IDEF2* uORF repressive function was conserved in other monocots we tested the *TaIDEF2-A1* 5’ LS using a DLA (Fig. 5B). The *TaIDEF2-A1* 5’ LS contains two exons (E1 and E2), and the uORF-containing region was located near the 5’ end of E2. All mutated sequences without the predicted uORF-containing region resulted in a large derepression of the mORF relative to WT sequence: 4.16-fold for *TaIDEF2* E1 (p_adjusted_ = < 0.001), 4.78-fold for *TaIDEF2* m3 (p_adjusted_ = < 0.001), and 4.41-fold for *TaIDEF2* m4 (p_adjusted_ = < 0.001), indicating one or several translationally repressive uORFs were severely disrupted. Smaller mutations in *TaIDEF2* m1 (1 bp deletion) and *TaIDEF2* m2 (ATG deletion) resulted in a 2.06-fold (p_adjusted_=0.514) and 2.79-fold (p_adjusted_=0.058) derepression of the mORF relative to the WT sequence, respectively. The predicted peptide from the uORF in the *TaIDEF2* m1 sequence was 7 aa shorter and had different peptide composition relative to the WT sequence, suggesting that peptide length and sequence is important for translational repression. The derepression of the *TaIDEF2* m3 sequence was 1.71-fold higher than the *TaIDEF2* m2 sequence (p_adjusted_=0.028), suggesting that other ribosomal binding sites or multiple uORFs may be present in the 5’ LS. *Nicotiana benthamiana* leaf segments infiltrated with the *TaIDEF2* m3 sequence exhibited elevated luminescence compared to *TaIDEF2* WT sequence (Fig. 5C). Similarly, in bread wheat (*Triticum aestivum* L.) leaves, the *TaIDEF2* m3 sequence exhibited a 1.3-fold higher luminescence than the *TaIDEF2* WT sequence (*p* = 0.03), inferred by the mean grey pixel values 36.4 and 47.8 for *TaIDEF2* WT and *TaIDEF2* m3, respectively (Fig. S7).

## Discussion

### Ribo-Seq datasets are useful for predicting peptide and non-peptide conserved uORFs

Several homology-based methods exist to identify uORFs (uORFinder, uORFSCAN, BAIUCAS/ESUCA) ([28, 32–35]. However, uORFs that are recently evolved and have low sequence conservation and non-conserved peptide uORFs (the largest group of uORFs) escape detection when analysing conserved non-canonical amino acid sequences across species. The development of ribosomal profiling, which allows the detection of cryptic translation events, has been particularly useful for uORF identification (Fig. S1). But predicting the exact start site of uORFs from ribo-seq datasets is challenging particularly as uORFs can contain non-canonical start codons which could be present in any frame [46]. The three-nucleotide periodicity of elongating ribosomes can theoretically be leveraged to determine the exact P-site of the 80S ribosome and thus the translation frame and translated peptide, however single nucleotide resolution ribo-seq datasets are difficult to produce for a number of experimental reasons [47]. Instead, we used ribosomal profiling to estimate the approximate location of uORFs followed by sequence and conservation analysis of all possible ORFs with non-canonical start sites (i.e. stop codon to stop codon). By contrast, the psORF platform predicts the exact start sites of uORFs using multiple ribo-seq datasets with low success likely due to noise and low-quality data (Fig. 2) [32]. For example, the psORF predicted *OsIDEF1* translation start site (CTG) resided in the middle of the 5’ LS, whereas our ribosomal profile of *OsIDEF1* indicated that translation was occurring towards the 5’ end (Fig. 1C). Further sequence analysis identified two canonical start sites (ATG) at the 5’ end and two at the 3’ end of the LS, with the second ATG at the 5’ end being responsible for significant mORF repression and the other ATGs being mostly redundant (Fig. 4). The second ATG has a 9 bp reading frame that begins 7 bp downstream of the 5’ cap and 265 bp upstream of the mORF start site. Corroborating our ribosomal profile (Fig. 1D) and conservation analysis (Fig. 5A, S6), the psORF platform identified the same ATG uORF start sites within the *OsIDEF2* 5’ LS that were found through ribosomal profiling. By contrast, the CTG start site (Fig. 1D) is unlikely to be a true uORF start site due to low sequence conservation amongst monocots (Fig. 5A). Thus, future research projects aimed at identifying uORFs should utilise the psORF platform alongside functional DLA testing to determine their presence, location, and start site (Fig S1C). The presence of uORFs with different reference frames in the *IDEF2*-uORF region cannot be excluded, although the main translationally repressive uORF is likely to contain a more favourable sequence context (i.e. an ATG start site) (Fig. 5A). In all analysed monocots, we detected an ATG-starting *IDEF2*-uORF that is 90 bp in length and has 29 highly conserved amino acids (Fig. 5A, S6). In general, this uORF appears 20 bp downstream from the 5’ end of a second exon present in most monocot *IDEF2* 5’ LS and approximately 529 bp upstream of the mORF start site. Interestingly, two small peaks are present within this ~ 529 bp region in the *OsIDEF2* ribosomal profile (Fig. 1D), which has low conservation amongst monocots (74.3% pairwise identity) relative to the ATG-starting uORF (92.6% pairwise identity). The DLA comparing *TaIDEF2* WT E1 (without the ~ 529 sequence) and *TaIDEF2* m4 (with the ~ 529 sequence) showed the same level of derepession, suggesting that the two small extra ribosomal peaks are either noise or weak non-conserved uORFs present only in the *OsIDEF2* 5’LS (Fig. 5B). Similarly, we detected moderate ribosomal peaks in the 5’ LS region of seven other Fe homeostasis genes analysed in this study, and DLA experiments will ultimately be required to confirm uORF presence and function in these genes.

### The peptide conserved *IDEF2*-uORF likely regulates Fe homeostasis in monocots

We were able to identify two Fe homeostasis regulatory genes with translationally repressive uORFs (*IDEF1* and *IDEF2*). The *IDEF1* and *IDEF2* genes encode for members of the ABI3/VP1 and NAC transcription factor families, respectively, and positively regulate the Fe deficiency response by binding to iron-deficiency-responsive elements 1 or 2 (IDE1 and IDE2) [20–22]. Under an Fe deficiency inducible promoter (IDS2p), the *OsIDEF1* gene increased tolerance to Fe deficiency in rice plants, however constitutive *IDEF1* expression causes poor growth under Fe sufficiency [21]. Knockdown of *OsIDEF2* by RNA interference and CRES-T increased sensitivity to Fe deficiency through an attenuated Fe deficiency response and lower expression of target genes such as *OsYSL2* [20]. The *OsIDEF1* and *OsIDEF2* genes are constitutively expressed during vegetative and reproductive growth stages in rice and expression remains relatively unchanged in root tissues under varying environmental Fe conditions [24]. Moreover, the expression of *TaIDEF2* is unchanged in wheat plants grown under Fe sufficient or deficient conditions [25]. Under high environmental Fe, the 5’ LSs from *OsIDEF2* and *OsIMA1* decreased translation of the mORF (Fig. 3, S3), suggesting that these regulatory regions respond to environmental Fe conditions. Further investigation revealed the *IDEF2* 5’ LS contains a translationally repressive CPuORF, which may regulate the mORF under high environmental Fe (Figs. 3 and 5). Interestingly, this phenomenon was also observed with the mutated *OsIDEF2* m1 sequence, suggesting that the *OsIDEF2* m1 sequence contains additional regulatory elements other than a translationally repressive uORF, or that the m1 mutation did not completely abolish the full function of the uORF. Regardless, these results provide mechanistic insight into the role of *IDEF2* in regulating the Fe deficiency response without alterations to transcription. The contrast in firefly luminescence between leaf segments infiltrated with WT *OsIDEF1*, *OsIDEF2*, and *TaIDEF2* sequences, and those infiltrated with mutated sequences provides functional *in planta* evidence of transient uORF repression and derepression (Figs. 2B and C and 5C, S7B). Further studies are now required to produce and assess heritable mutations in monocots. Additional investigation is also required to elucidate how the *IDEF2*-uORF dynamically represses the mORF and whether it functions as a secondary metabolite and/or directly interacts with Fe. The non-peptide conserved *OsIDEF1*-uORF identified in this study could play a role in regulating the Fe deficiency response but is unlikely to be the primary pathway for upregulating downstream genes. The *OsIDEF1*-uORF did not respond to external Fe changes and is not conserved within monocots (Fig. 4, S4). The OsIDEF1 transcription factor interacts directly with Fe ions, which is likely to be the main post-transcriptional mechanism by which OsIDEF1 regulates the Fe deficiency response [23]. Despite this, increasing *OsIDEF1* expression through precision editing of the *OsIDEF1*-uORF could result in positive impacts on rice abiotic stress tolerance and nutrition and will be the subject of future studies.

### Regulating gene expression through uORF modification could be applied to future plant breeding

The world population is expected to reach 9.7 billion by 2050 and global crop production needs to increase between 35 and 56% to meet the growing demand for food [48]. Site directed nuclease 1 (non-homologous end-joining) CRISPR-Cas9 methods are effective at gene knockouts, but precise increases and decreases to gene expression are more challenging. Upstream ORFs can be exploited for fine-tuning of target gene expression, such as the use of base and prime editing to knock in de novo uORFs into rice resulting in highly predictable reductions in gene translation [49]. Our *TaIDEF2*-uORF mutation series (Fig. 5) supports the ability to incrementally increase gene translation through step-wise mutations of pre-existing uORFs, and the identification of uORFs in *IDEF* genes demonstrates the utility and practicality of our uORF identification workflow (Fig. S1). Together these techniques represent powerful tools to enable understanding of post-transcriptional regulation in plant Fe homeostasis and identify novel targets for future crop improvement.

## Electronic supplementary material

Below is the link to the electronic supplementary material.

Supplementary Material 1

Supplementary Material 2

Supplementary Material 3

Supplementary Material 4

Supplementary Material 5: Figure S1: Summary of uORF identification and validation in plant genes. Figure S2: Ribosomal profiles of 27 rice transcripts and one Arabidopsis transcript to detect non-canonical translation in the 5’ LS. Figure S3: Dual luciferase assay of the *OsIMA1* 5’ LS under control conditions and increased environmental Fe. Figure S4: Dual luciferase assay and conservation of the *TaIDEF1*-B 5’ LS. Figure S5: Replicated and independently infiltrated *N. benthamiana* containing the *TaIDEF2* WT and m3 5’ LS. Figure S6: Alignment and phylogeny of the *IDEF2*-uORF region in monocots. Figure S7: Visual representation of independently infiltrated bread wheat (*Triticum aestivum* L.) leaves containing various dual luciferase constructions.

## Data Availability

All relevant data including raw data is presented in this manuscript and supplementary information files.

## Abbreviations

CPuORF: Conserved Peptide upstream Open Reading Frame
DLA: Dual luciferase assay
Fe: Iron
LS: Leader sequence
uORF: Upstream Open Reading Frame
WT: Wild-type
