# Supplementary material for: Discovery of a conserved translationally repressive upstream open reading frame within the iron-deficiency response regulator IDEF2
Source: BMC Plant Biol. 2024 Sep 30;24:891. doi: 10.1186/s12870-024-05473-y (PMC11440899; doi:10.1186/s12870-024-05473-y)
Supplement: Supplementary file 5 — Supplementary Material 5: Figure S1: Summary of uORF identification and validation in plant genes. Figure S2: Ribosomal profiles of 27 rice transcripts and one Arabidopsis transcript to detect non-canonical translation in the 5’ LS. Figure S3: Dual luciferase assay of the OsIMA1 5’ LS under control conditions and increased environmental Fe. Figure S4: Dual luciferase assay and conservation of the TaIDEF1-B 5’ LS. Figure S5: Replicated and independently infiltrated N. benthamiana containing the TaIDEF2 WT and m3 5’ LS. Figure S6: Alignment and phylogeny of the IDEF2-uORF region in monocots. Figure S7: Visual representation of independently infiltrated bread wheat (Triticum aestivum L.) leaves containing various dual luciferase constructions. [file 12870_2024_5473_MOESM5_ESM.docx]

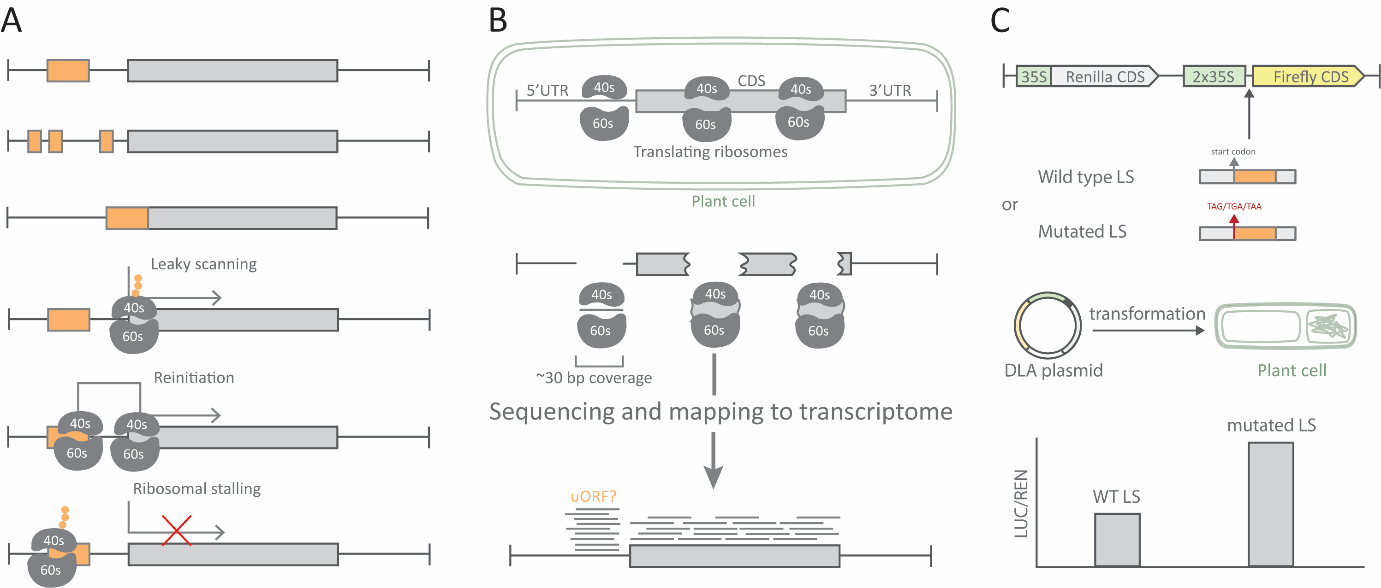


Figure S1: Summary of uORF identification and validation in plant genes. (A) Schematic showing the diversity of uORFs. Single or multiple uORFs can exist in a 5’ LS and can overlap with the main open reading frame. The mechanism that uORFs regulate translation include leaky scanning, reinitiation, and ribosomal stalling. The transcripts are represented with the 5’ LS (left-hand horizontal line), coding sequence (grey box), 3’ UTR (right-hand horizontal line), and uORFs (orange box). (B) Schematic showing the use of ribosomal profiling to identify regions of non-canonical translation. Translating ribosomes are extracted from plant cells, unprotected RNA is digested, and 30 bp ribosomal covered regions are sequenced and mapped back to the transcriptome to identify putative uORFs. (C) Schematic showing how a DLA can validate putative uORFs. The dual luciferase construct contains the renilla luciferase coding sequence (internal control) and firefly luciferase coding sequence (reporter). A WT or mutated 5’ LS is cloned upstream of the firefly coding sequence and the dual luciferase construct is transformed into a plant cell. Increased luminosity within the mutated construct validates the presence of a translationally repressive uORF.

Table S1: List of 20 genes and their splice variants with ribosomal data analysed in this study.

| **Gene name** | **Transcript ID** | **Splice variant** | **LOC ID** | **Average ribosomal coverage** | **5' LS ribosomal peaks** |
| --- | --- | --- | --- | --- | --- |
| *OsDMAS1* | Os03t0237100-01 | 1 | LOC_Os03g13390 | 3.549 | no |
| *OsFER1* | Os11t0106700-01 | 1 | LOC_Os11g01530 | 58.877 | no |
| *OsFER1* (2) | Os11t0106700-02 | 2 | LOC_Os11g01530 | 67.585 | no |
| *OsFER2* | Os12t0106000-01 | 1 | LOC_Os12g01530 | 137.781 | no |
| *OsHRZ1* | Os01t0689451-01 | 1 | n/a | 3.11 | weak |
| *OsHRZ1* (2) | Os01t0689451-02 | 2 | n/a | 3.459 | weak |
| *OsHRZ2* | Os05t0551000-01 | 1 | LOC_Os05g47780 | 2.705 | weak |
| *OsIDEF1* | Os08t0101000-01 | 1 | LOC_Os08g01090 | 9.64 | medium |
| *OsIDEF2* | Os05t0426200-02 | 1 | LOC_Os05g35170 | 20.089 | strong |
| *OsIMA1* | Os01t0647200-01 | 1 | LOC_Os01g45914 | 5.187 | weak |
| *OsIMA2* | Os07t0142100-00 | 1 | n/a | 6.939 | no |
| *OsIRO2* | Os01t0952800-01 | 1 | LOC_Os01g72370 | 1.246 | weak |
| *OsNAS3* | Os07t0689600-01 | 1 | LOC_Os07g48980 | 10.622 | no |
| *OsPRI1* | Os08t0138500-01 | 1 | LOC_Os08g04390 | 14.857 | no |
| *OsPRI2* | Os05t0455400-01 | 1 | LOC_Os05g38140 | 19.328 | weak |
| *OsPRI3* | Os02t0116600-01 | 1 | LOC_Os02g02480 | 8.079 | no |
| *OsPRI3* (2) | Os02t0116600-01 | 2 | LOC_Os02g02480 | 8.663 | no |
| *OsYSL2* | Os02t0649900-01 | 1 | LOC_Os02g43370 | 1.299 | no |
| *OsYSL6* | Os04t0390500-01 | 1 | LOC_Os04g32050 | 4.965 | no |
| *OsYSL6* (2) | Os04t0390500-02 | 2 | LOC_Os04g32050 | 5.112 | no |
| *OsYSL6* (3) | Os04t0390500-03 | 3 | LOC_Os04g32050 | 5.68 | no |
| *OsYSL6* (4) | Os04t0390500-04 | 4 | LOC_Os04g32050 | 5.551 | no |
| *OsYSL9* | Os04t0542200-01 | 1 | LOC_Os04g45860 | 5.186 | no |
| *OsYSL12* | Os04t0524600-01 | 1 | LOC_Os04g44320 | 1.938 | no |
| *OsYSL16* | Os04t0542800-01 | 1 | LOC_Os04g45900 | 7.34 | medium |
| *OsGGP* | Os12t0190000-01 | 1 | LOC_Os12g08810 | 14.594 | strong |
| *OsGGP* (2) | Os12t0190000-02 | 2 | LOC_Os12g08810 | 8.49 | no |

The above genes and splice variants represent a subset of Fe homeostasis transcripts that contained sufficient ribosomal coverage to generate ribosomal profiles. The gene IDs were extracted from FunRiceGenes and the strength of ribosomal peaks in the 5’ LS were determined through the assessment of self-contained peaks in the 5’ LS of ribosomal profiles in Fig. S2.


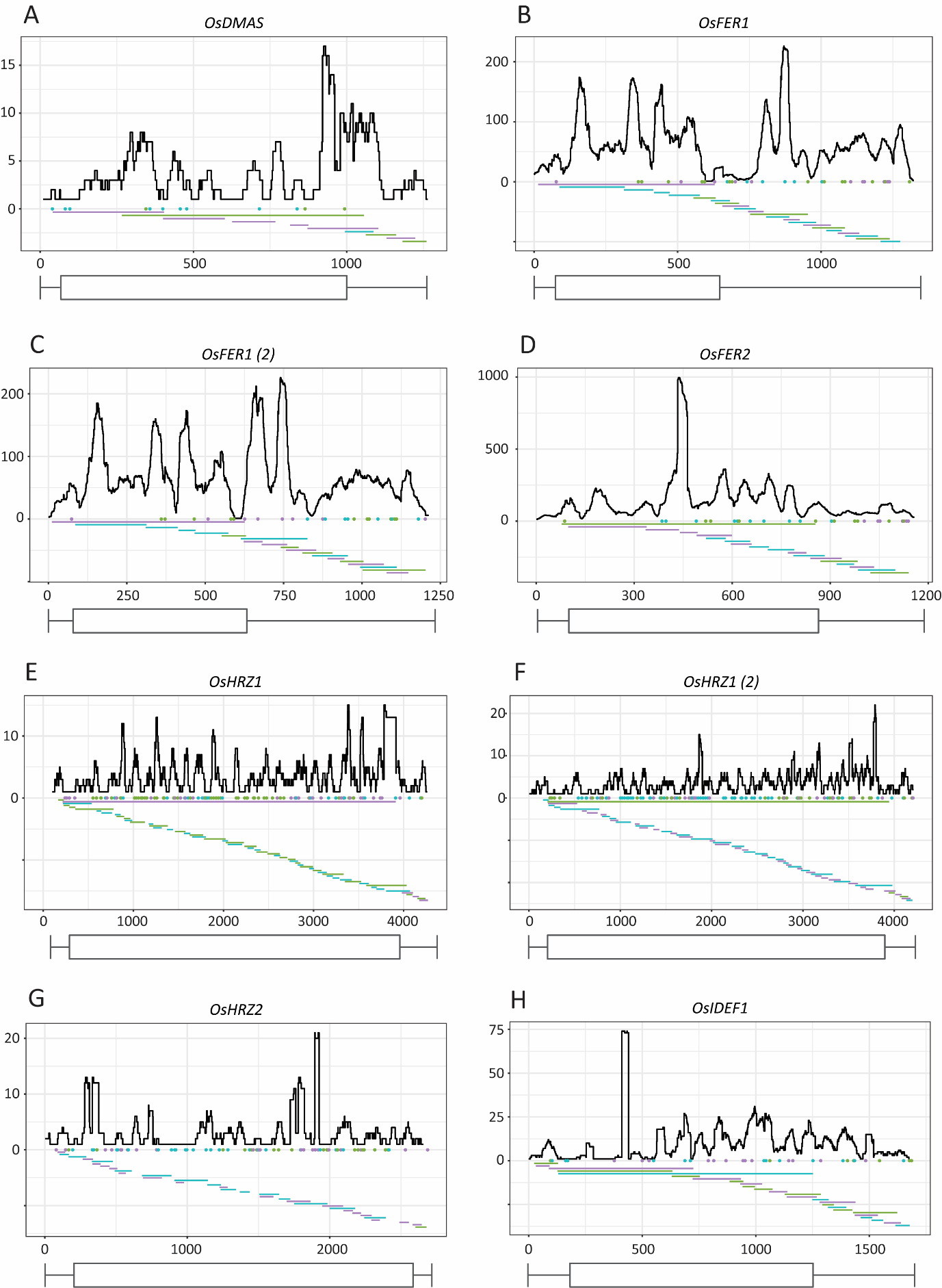


Figure S2 cont.


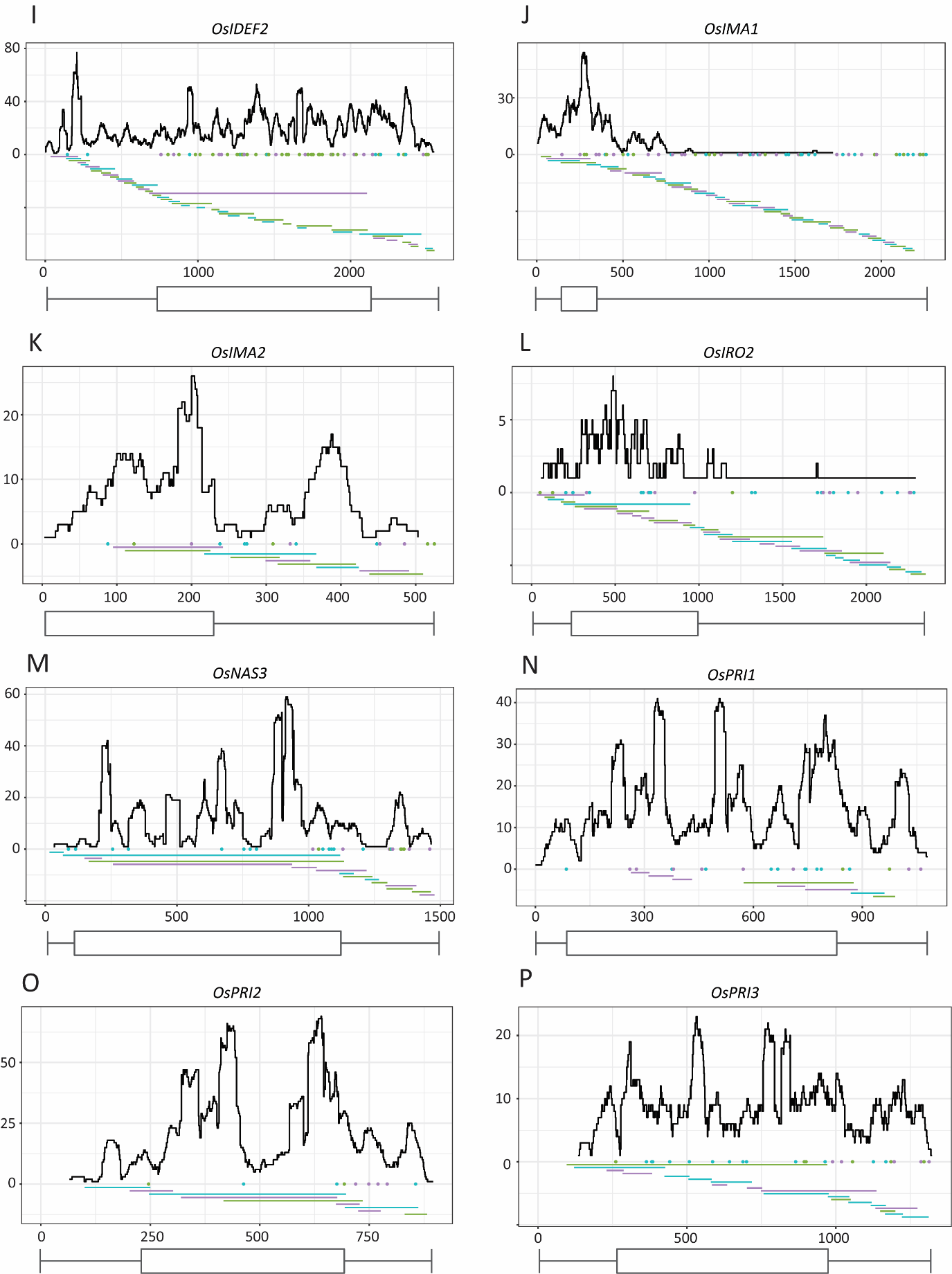


Figure S2 cont.


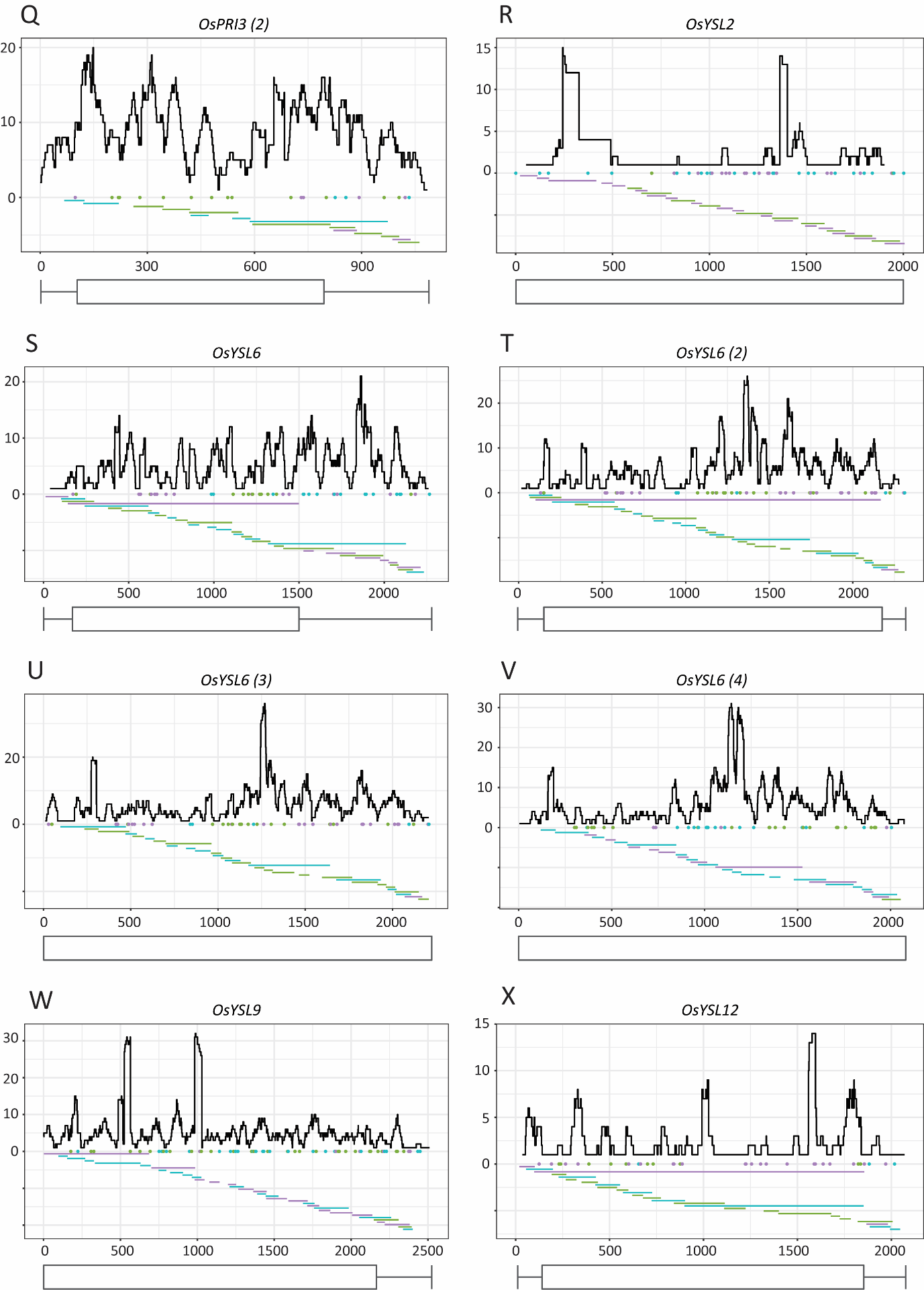


Figure S2 cont.


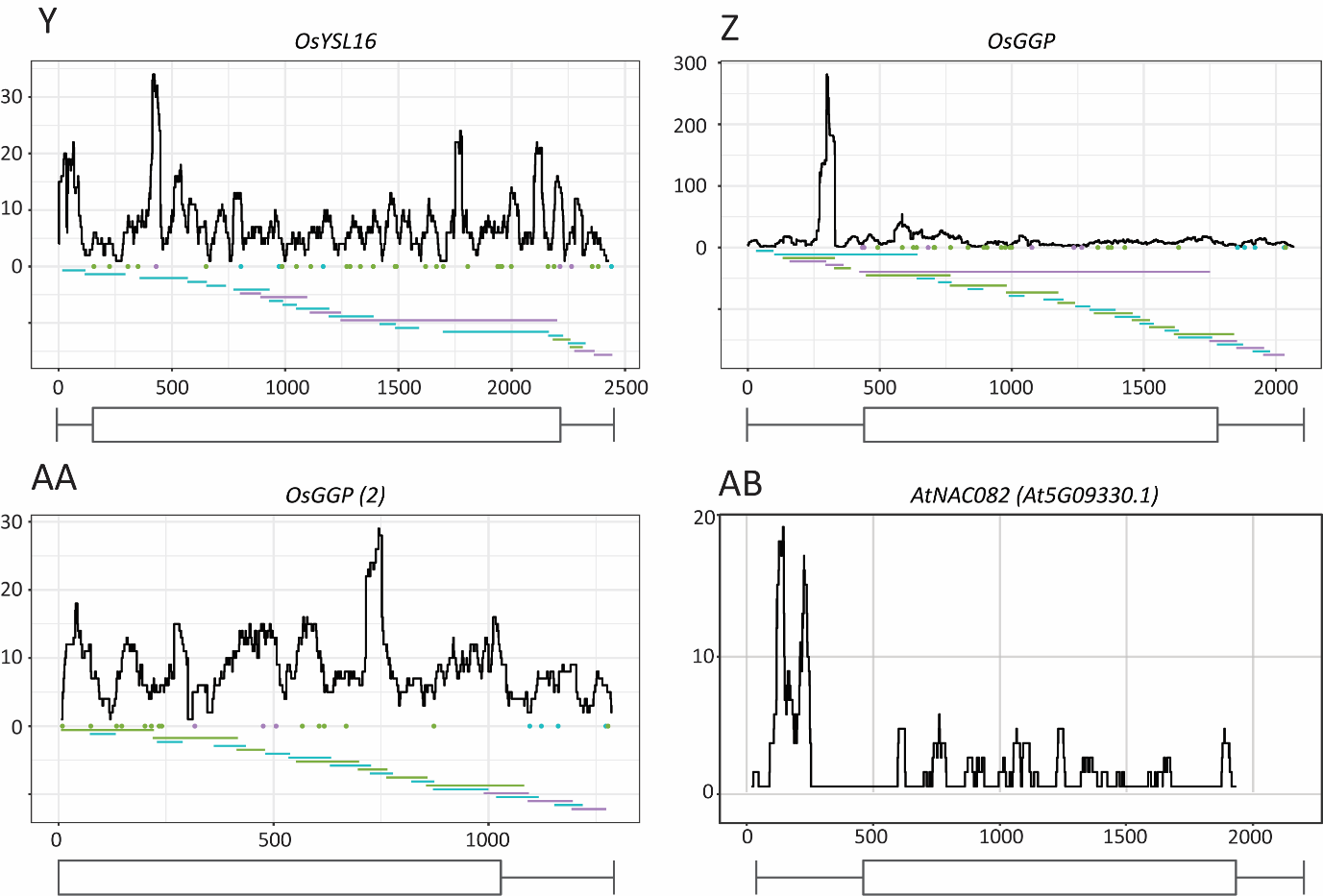


Figure S2: Ribosomal profiles of rice (A-AA) and one *Arabidopsis thaliana* transcript (AB) involved in nutrient homeostasis. Canonical start codons within translation frame 1 (green), 2 (blue), and 3 (purple) are represented as dots along the x-axis. Stop-to-stop ORFs are indicated below the x-axis within translation frame 1 (green), 2 (blue), and 3 (purple). The transcript models are represented below the ribosomal profile with the 5’ LS (left-hand horizontal line), coding sequences (coloured boxes), and 3’ UTR (right-hand horizontal line).


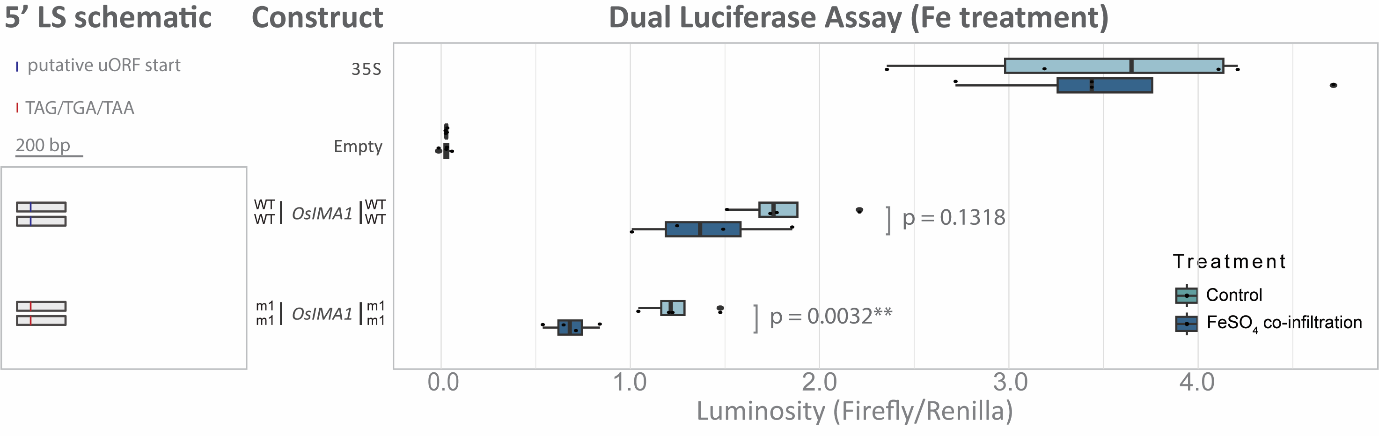
 Figure S3: Dual luciferase assay of the *OsIMA1* 5’ LS from rice. Schematic (left panel) of the WT or mutated (m1) 5’ LS that were fused upstream of the firefly luciferase coding sequence and downstream of a 35S promoter. A box plot (right panel) compares luminosity ratios (firefly/renilla) of the various 5’ LS as either the control single infiltrations (light blue) or co-infiltrations with FeSO_4_ (dark blue) to perturb Fe homeostasis. The p-values compare the control and co-infiltrated 5’ LS as determined by a two-sample Students t-test assuming unequal variance (n=4). Each biological replicate comprised of three leaf discs (averaged) from three infiltration sites on a single leaf. The luminosity ratios (firefly/renilla) were not normalised to the 35S vectors to demonstrate that FeSO_4_ co-infiltration does not interact the luciferase genes without the addition of Fe-related 5’ LS.


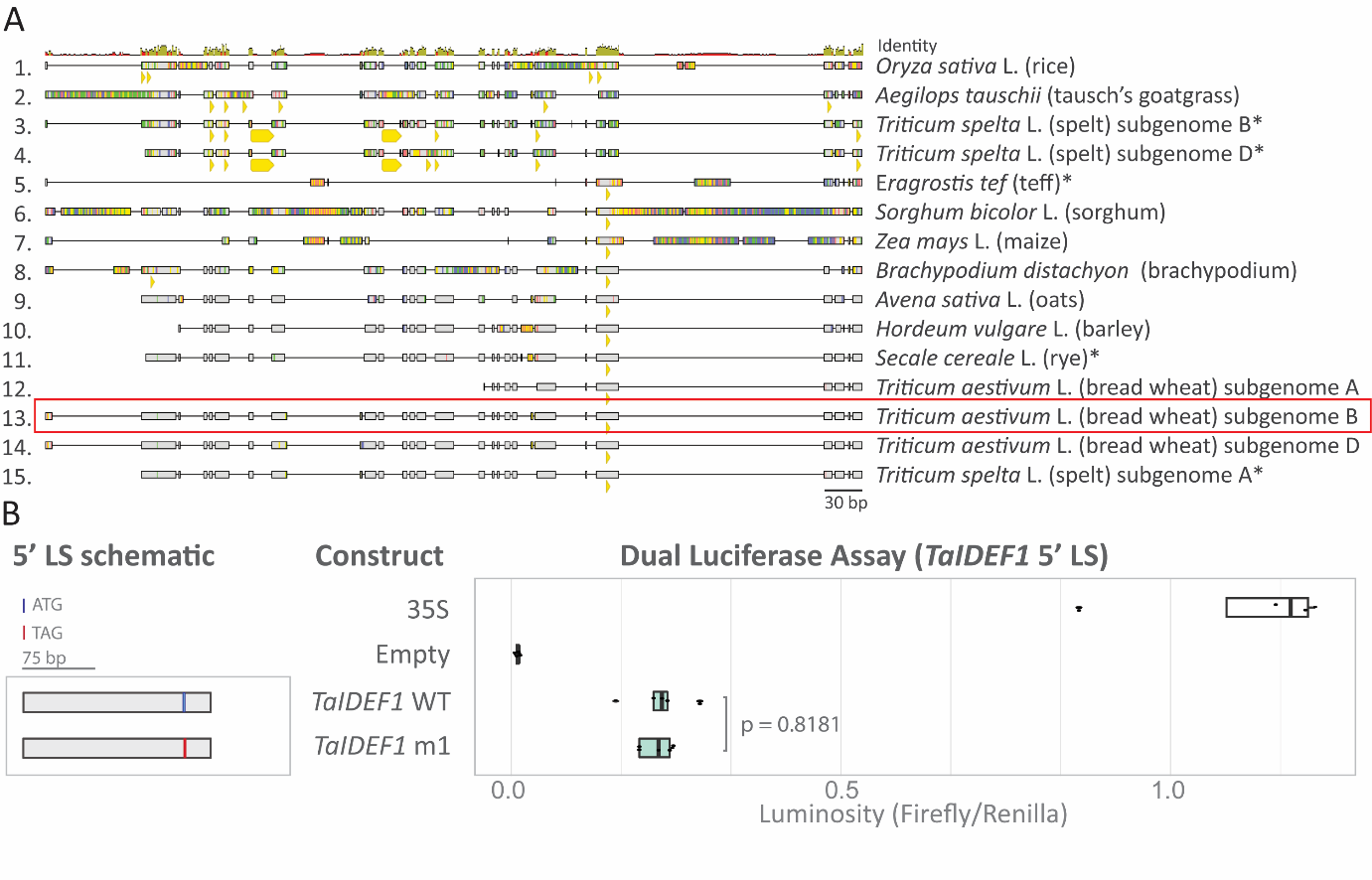
 Figure S4: Dual luciferase assay of the *TaIDEF1-B1* 5’ LS from bread wheat. (A) Nucleotide alignment of 5’ LS amongst *IDEF1* orthologous sequences in monocots. A semi-conserved canonical start codon (yellow arrow) is indicated as a possible uORF start site. The red rectangle indicates the 5’ LS used for the following DLA. (B) Schematic (left panel) of the WT or mutated 5’ LS that were fused upstream of the firefly luciferase coding sequence and downstream of a 35S promoter. A box plot (right panel) compares luminosity ratios (firefly/renilla) of the mutated or WT 5’ LS. The p-value compares the WT and mutated 5’ LS as determined by a two-sample Students t-test assuming unequal variance (n=5). Each biological replicate comprised of three leaf discs from three infiltration sites on a single leaf. The luminosity ratios (firefly/renilla) were normalised to the 35S vector (positive control containing no 5’ LS).


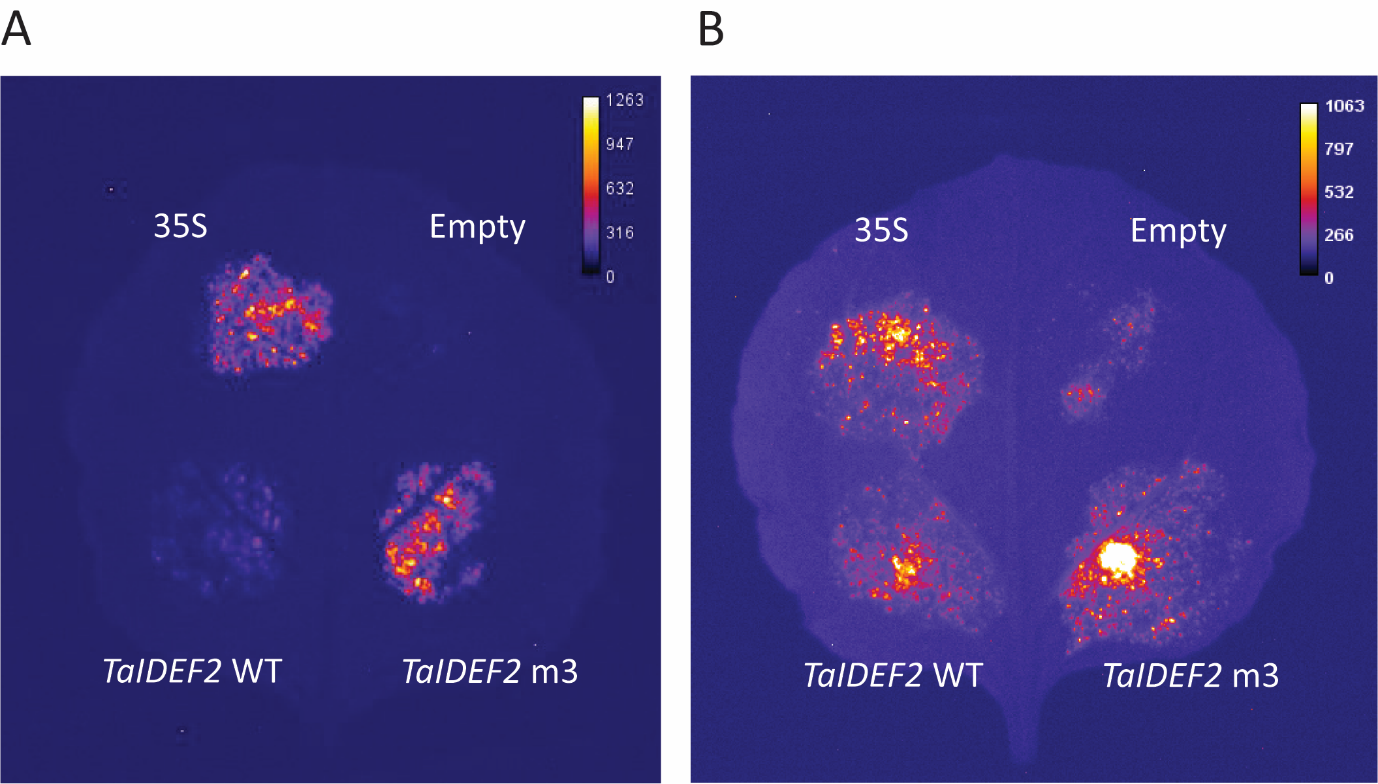
 Figure S5: (A, B) Visual representation of replicated and independently infiltrated *N. benthamiana* leaves containing the 35S infiltration (top left), empty vector infiltration (top right), *TaIDEF2* WT (bottom left) and *TaIDEF2* m3 (bottom right).


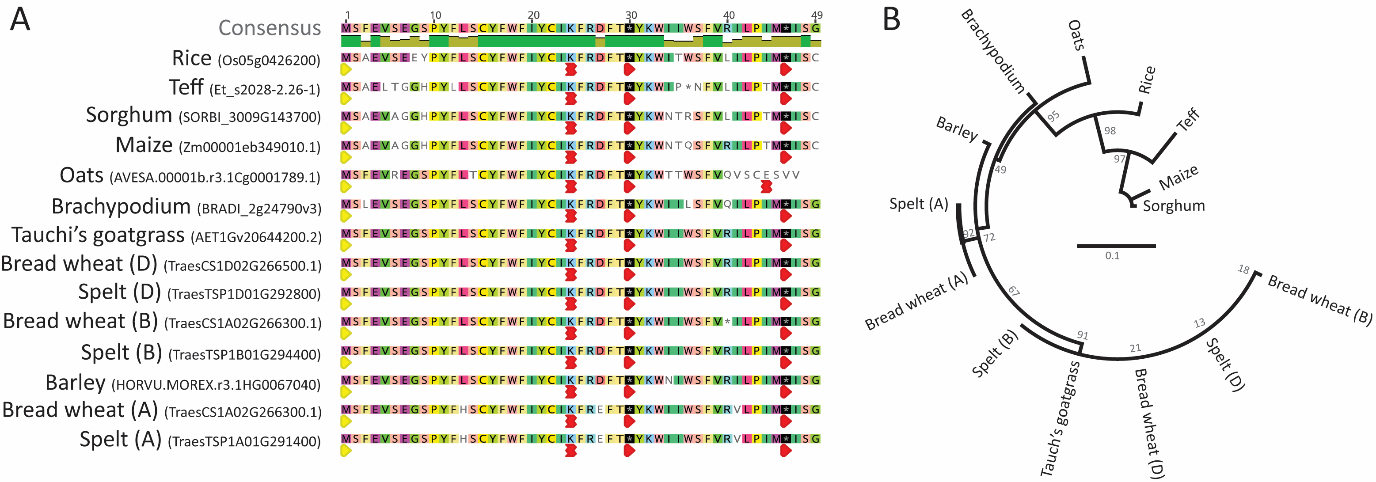


Figure S6: Alignment and phylogeny of the *IDEF2*-uORF region in monocots. (a) Protein alignment of a translation frame in the *IDEF2*-uORF region with a canonical start site indicated (yellow arrow) and several stop codons indicated (red arrows) in the same or different frames. (b) Circular phylogenetic tree generated from a coding sequence alignment of *IDEF2*-uORF regions in monocots. The numbers at each node indicate the bootstrap proportion and the scale bar represents the number of substitutions per site.


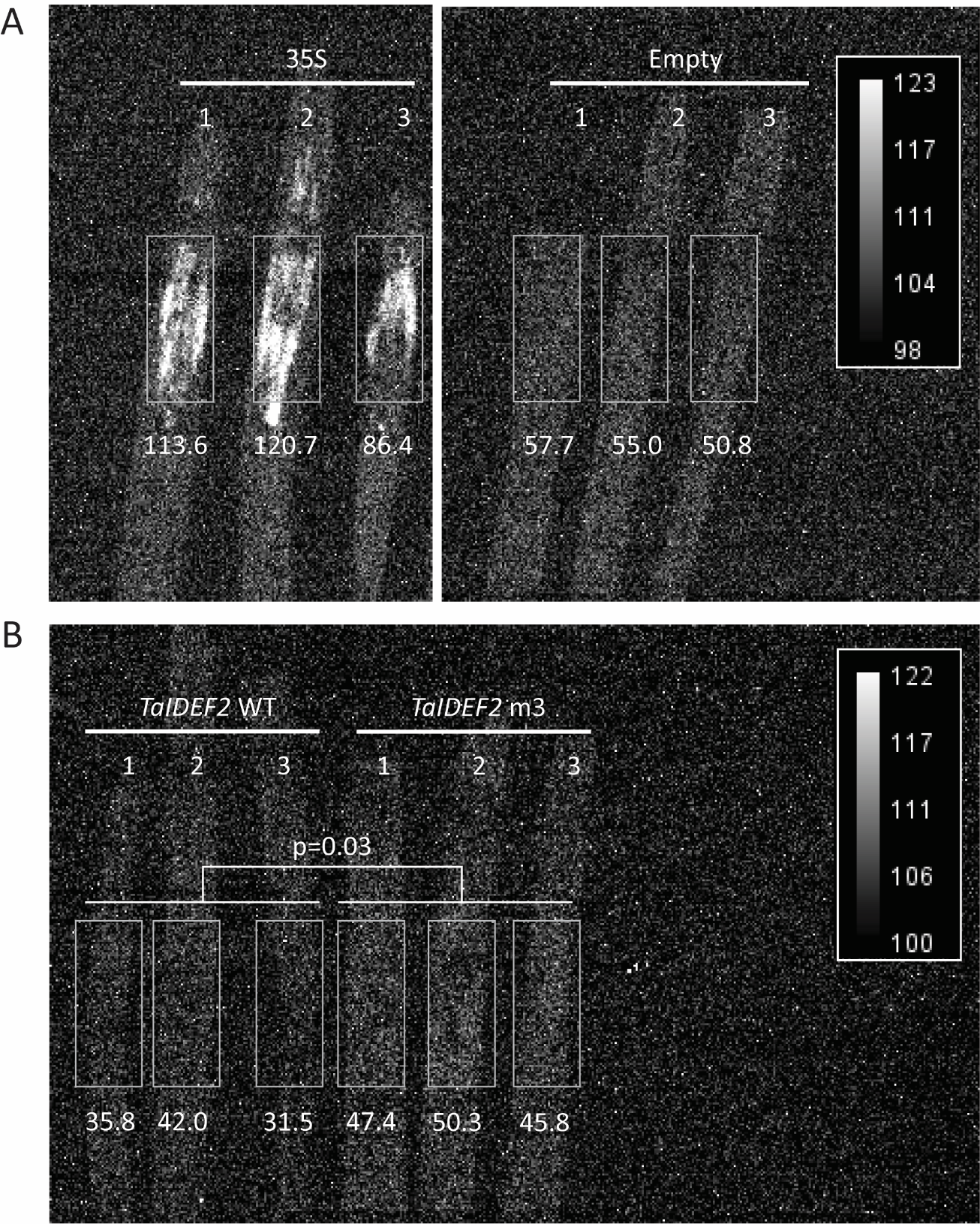


Figure S7: Visual representation of infiltrated bread wheat (*Triticum aestivum* L.) leaves containing (a) the 35S infiltration (left), empty vector infiltration (top right), (b) *TaIDEF2* WT (left) and *TaIDEF2* m3 (right). The rectangles represent and area of 2496 pixels and the numbers below represent the mean grey value inside the rectangle. The p-value comparing the mean of the three *TaIDEF1* WT infiltrations and the three *TaIDEF2* m3 infiltrations was determined using a Student’s Two-tailed T-test assuming equal variance.
